# SARS-CoV-2 Antibody Rapid Tests: Valuable Epidemiological Tools in Challenging Settings

**DOI:** 10.1128/Spectrum.00250-21

**Published:** 2021-09-22

**Authors:** Francesca Saluzzo, Paola Mantegani, Valeria Poletti De Chaurand, Virginia Quaresima, Federica Cugnata, Clelia Di Serio, Aurélien Macé, Margaretha De Vos, Jilian A. Sacks, Daniela Maria Cirillo

**Affiliations:** a Division of Immunology, Transplantation and Infectious Disease, IRCCS Ospedale San Raffaele, Milano, Italy; b CUSSB-University Center for Statistics in the Biomedical Sciences, Vita-Salute San Raffaele University, Milano, Italy; c Foundation for Innovative New Diagnostics (FIND), Geneva, Switzerland; Karolinska Institutet

**Keywords:** SARS-CoV-2 immunology, cross-reactivity, lateral flow assays, low-middle-income countries, performance, point-of-care tests

## Abstract

During the last year, mass screening campaigns have been carried out to identify immunological response to severe acute respiratory syndrome coronavirus 2 (SARS-CoV-2) and establish a possible seroprevalence. The obtained results gained new importance with the beginning of the SARS-CoV-2 vaccination campaign, as the lack of doses has persuaded several countries to introduce different policies for individuals who had a history of COVID-19. Lateral flow assays (LFAs) may represent an affordable tool to support population screening in low-middle-income countries, where diagnostic tests are lacking and epidemiology is still widely unknown. However, LFAs have demonstrated a wide range of performance, and the question of which one could be more valuable in these settings still remains. We evaluated the performance of 11 LFAs in detecting SARS-CoV-2 infection, analyzing samples collected from 350 subjects. In addition, samples from 57 health care workers collected at 21 to 24 days after the first dose of the Pfizer-BioNTech vaccine were also evaluated. LFAs demonstrated a wide range of specificity (92.31% to 100%) and sensitivity (50% to 100%). The analysis of postvaccination samples was used to describe the most suitable tests to detect IgG response against S protein receptor binding domain (RBD). Tuberculosis (TB) therapy was identified as a potential factor affecting the specificity of LFAs. This analysis identified which LFAs represent a valuable tool not only for the detection of prior SARS-CoV-2 infection but also for the detection of IgG elicited in response to vaccination. These results demonstrated that different LFAs may have different applications and the possible risks of their use in high-TB-burden settings.

**IMPORTANCE** Our study provides a fresh perspective on the possible employment of SARS-CoV-2 LFA antibody tests. We developed an in-depth, large-scale analysis comparing LFA performance to enzyme-linked immunosorbent assay (ELISA) and electrochemiluminescence immunoassay (ECLIA) and evaluating their sensitivity and specificity in identifying COVID-19 patients at different time points from symptom onset. Moreover, for the first time, we analyzed samples of patients undergoing treatment for endemic poverty-related diseases, especially tuberculosis, and we evaluated the impact of this therapy on test specificity in order to assess possible performance in TB high-burden countries.

## INTRODUCTION

An accurate knowledge of the local epidemiology of severe acute respiratory syndrome coronavirus 2 (SARS-CoV-2) has proven itself crucial to managing the different phases of the COVID-19 pandemic. The possibility to rely on consistent epidemiological data may be useful in making several public health decisions also related to the COVID-19 vaccination campaign and the World Health Organization (WHO) COVID-19 Vaccines Global Access (COVAX) program (1, 2). In the current scenario, in which the lack of vaccine doses has persuaded several countries to introduce different policies for individuals who had a history of SARS-CoV-2 infection (a decision that has not been fully endorsed by WHO) (3), access to reliable data about the serological status of individuals could gain new importance (4). However, whereas serological mass screening in high-income countries could be feasible using automatic, high-throughput technologies (5, 6), this may not be a practical option in several challenging diagnostic settings where the prevalence of SARS-CoV-2 infection is still widely unknown (7). In these countries, lateral flow assays (LFAs) for the identification of SARS-CoV-2 antibodies may represent an affordable and practical tool to perform epidemiological evaluations, but only a few serological surveys have been conducted to date employing LFAs, associated or not with enzyme-linked immunosorbent assays (ELISA) (8, 9).

Even if several studies have demonstrated their variable performance (10–13), SARS-CoV-2 antibody detection LFAs are currently considered a homogeneous group with comparable specificity and sensitivity. This fact contributed to a common feeling of distrust toward them in the scientific community (14), and a consensus on which LFA could be employed as an effective epidemiological tool has not been reached. To date, WHO recommends their use only in research for possible epidemiological employment (15, 16), and no LFA has received a WHO emergency use listing.

An accurate evaluation of which LFA offers the highest reliability in identifying SARS-CoV-2 infected individuals or in monitoring the immune response to the vaccine could be helpful to select an effective and cheap tool to be used in challenging diagnostic settings.

In this regard, our laboratory evaluated the performance of 11 different LFAs and one ELISA in detecting SARS-CoV-2 infection, analyzing plasma and serum samples collected from 350 subjects.

Moreover, to evaluate which tests could be useful in identifying an immune response developed in response to the COVID-19 vaccine, 57 plasma samples were collected at 21 to 24 days from the first dose of the BNT162b2 Pfizer-BioNTech vaccine. These samples were examined using the 11 different LFAs and an electrochemiluminescence immunoassay (ECLIA) dosing IgG against spike protein receptor binding domain (RBD).

## RESULTS

### Test performance.

Six out of the 11 analyzed LFAs demonstrated perfect specificity in healthy negative controls collected before 2019 (PreH) for both IgM and IgG. BTNX, QuickZen, and Tigsun identified one PreH sample as IgM positive (0.89%; 95% confidence interval [CI], 0.02 to 4.87); Perfectus and Tigsun identified two samples as IgG positive (1.75%; 95% CI, 0.21 to 6.19 and 1.77%; 95% CI, 0.22 to 6.25, respectively); and RightSign identified one as IgG positive (0.88%, 95% CI, 0.02 to 4.83).

**FIG 1:**
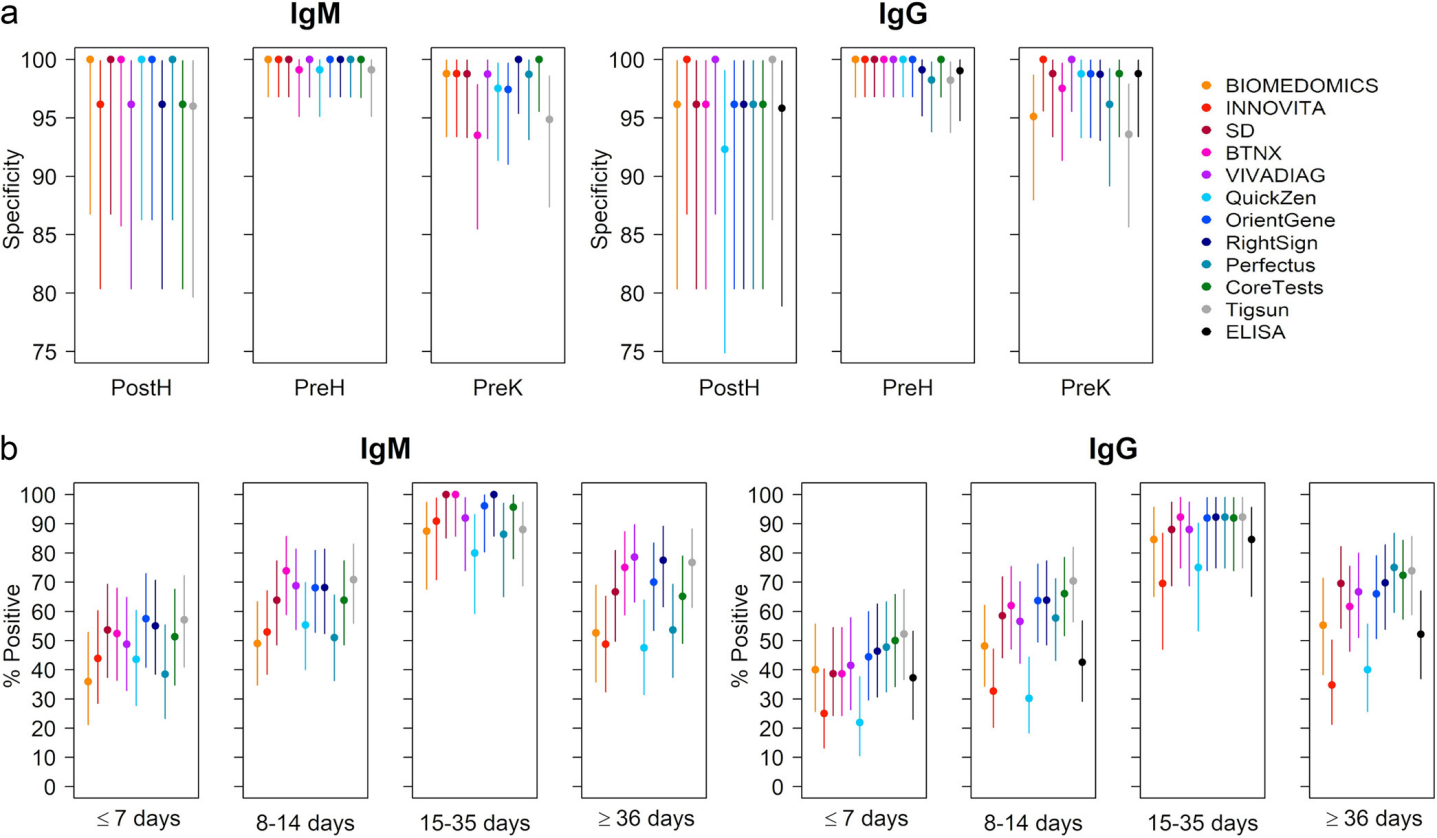
Test performance data plotted for IgG and IgM. (a) Specificity is calculated for each LFA and ELISA (only for IgG) using samples collected from healthy donors sampled before 2019 (PreH, *n* = 114), pre-COVID-19-negative samples collected from individuals in treatment for TB (PreK, *n* = 82), and samples collected from volunteers who tested negative to SARS-CoV-2 rRT-PCR performed on nasopharyngeal samples surveyed in 2020 (PostH, *n* = 26). (b) Percent of samples from COVID-19 patients (COVID-19 POS) that tested positive by each LFA and ELISA (only for IgG) plotted in relation to time after symptom onset. COVID-19 POS ≤ 7 days, *n* = 45; COVID-19 POS 8 to 14 days, *n* = 55; COVID-19 POS 15 to 35, *n* = 26; COVID-19 POS ≥ 36, *n* = 47. All nodes identify the determined specificity (a) or positivity percentage (b). Error bars refer to 95% CIs.

The number of false positives increased dramatically in the group of samples collected before 2019 from patients receiving therapy for tuberculosis (TB) (PreK). There was at least one indeterminate or false positive for IgM and/or IgG for these samples across all LFAs (Fig. 1a).

In the PostH cohort, LFA specificity ranged from 96.15% to 100% for IgM and from 92.31% to 100% for IgG. ELISA demonstrated a specificity of 95.83% (95% CI, 78.88 to 99.89).

Logistic mixed-effects models were used to evaluate the differences among PreH, PostH, and PreK groups.

For IgM, the analysis showed that the results were significantly less likely to be negative in tuberculosis subjects (PreK) compared to healthy subjects (PreH) (odds ratio [OR] = 0.12; *P* value = 0.0115) (see Table S2 in the supplemental material). For IgG, no statistically significant differences have been observed (see Table S3 in the supplemental material).

In the models, the effects of sex, age, and ethnicity were also assessed, and no differences have been observed. The group analyzed, including COVID-19 patients and negative controls, had a median age of 33 years old (from 18 to 84 years old); males and females were, respectively, 59% and 41%; 79.8% were Caucasian, 6.1% Hispanic, 4.3% Asian, and 9.8% black.

As different times of seroconversion have been reported in literature (17), the sensitivity of the tests was assessed in samples collected across different periods in relation to the occurrence of the symptoms. The results of the evaluation are shown in Fig. 1b.

Of the six tests that demonstrated perfect specificity in the PreH group (BioMedomics, Innovita, SD, VivaDiag, Orient Gene, and Coretests), Orient Gene showed the highest sensitivity for IgM at <7 days from symptoms onset (57.50%; 95% CI, 40.89 to 72.96) and Coretests for IgG (50%; 95% CI, 34.19 to 65.81). Between 8 and 14 days from symptoms onset, VivaDiag had the highest IgM sensitivity of 68.75% (95% CI, 53.75 to 81.34), and Coretests had the highest IgG sensitivity of 66.04% (95% CI, 51.73 to 78.48). At 15 to 35 days, SD had a sensitivity of 100% for IgM (95% CI, 85.18 to 100) and Coretests of 92.31% for IgG (95% CI, 73.97 to 99.02). Finally, VivaDiag still had a 78.57% IgM sensitivity at more than 36 days from symptoms onset (95% CI, 63.19 to 89.70), and Coretests identified 72.34% (95% CI, 57.36 to 84.38) of the samples in this group as IgG positive.

Overall, LFA capability of identifying individuals with a SARS-CoV-2 infection was proven by real-time reverse transcriptase PCR (rRT-PCR) performed on nasopharyngeal swab (NPS). Logistic mixed-effects models, followed by *post hoc* pairwise comparisons (Bonferroni-corrected for multiple comparisons), were used to evaluate the differences among groups defined according to the time of sampling. For IgM, significant differences emerged only between samples collected at ≤7 days from symptom onset and samples collected at 15 to 35 days (*P* = 0.0072) (see Table S4 in the supplemental material). For IgG, significant differences emerged between samples collected at ≤7 days from symptom onset and samples collected at 15 to 35 days (*P* = 0.0004) (see Table S5 in the supplemental material) as well as samples collected at 8 to 14 days from symptom onset and samples collected at 15 to 35 days (*P* = 0.0188) (see Table S5).

IgM and IgG kinetics in SARS-CoV-2 infection are still not completely understood (17). Our study confirmed the tendency of IgM and IgG to rise at the same time in COVID-19 patients, as well as a lower specificity of IgM in identifying the infected individuals (10, 17).

### Indeterminate analysis.

**FIG 2:**
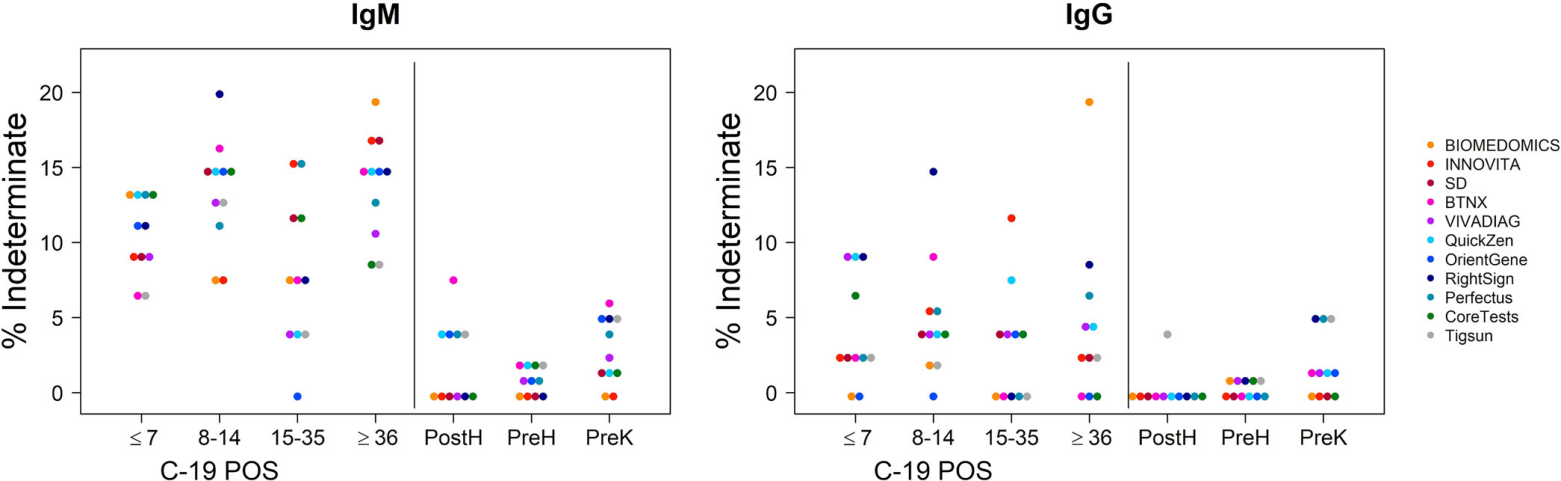
Indeterminate results identified by LFAs in the different groups in analysis. Indeterminate results plotted for IgM and IgG for each LFA in analysis. A total of 395 samples were analyzed to determine each LFA performance (179 from patients positive to rRT-PCR on NPS and 222 collected before 2019 or from patients negative to rRT-PCR on NPS). IgM-indeterminate results were reported more frequently than IgG. Total number of IgM-indeterminate results for each test is as follows: BioMedomics, 21/395; Innovita, 20/395; SD, 24/395; BTNX, 30/395; VivaDiag, 20/395; QuickZen, 26/395; Orient Gene, 26/395; RightSign, 29/395; Perfectus, 27/395; Coretests, 24/395; Tigsun, 22/395. Total number of IgG indeterminate results for each test is as follows: BioMedomics, 11/395; Innovita, 8/395; SD, 5/395; BTNX, 7/395; VivaDiag, 11/395; QuickZen, 11/395; Orient Gene, 2/395; RightSign, 21/395; Perfectus, 11/395; Coretests, 7/395; Tigsun, 9/395.

Indeterminate results were observed for all of the tests in the analysis. IgM indeterminate results were reported more frequently than IgG, and each indeterminate test was repeated once. After repetition, the two tests with the highest number of IgM indeterminate results were BTNX (30/395) and RightSign (29/395). RightSign also had the highest number of IgG indeterminate results (21/395) (Fig. 2).

**FIG 3:**
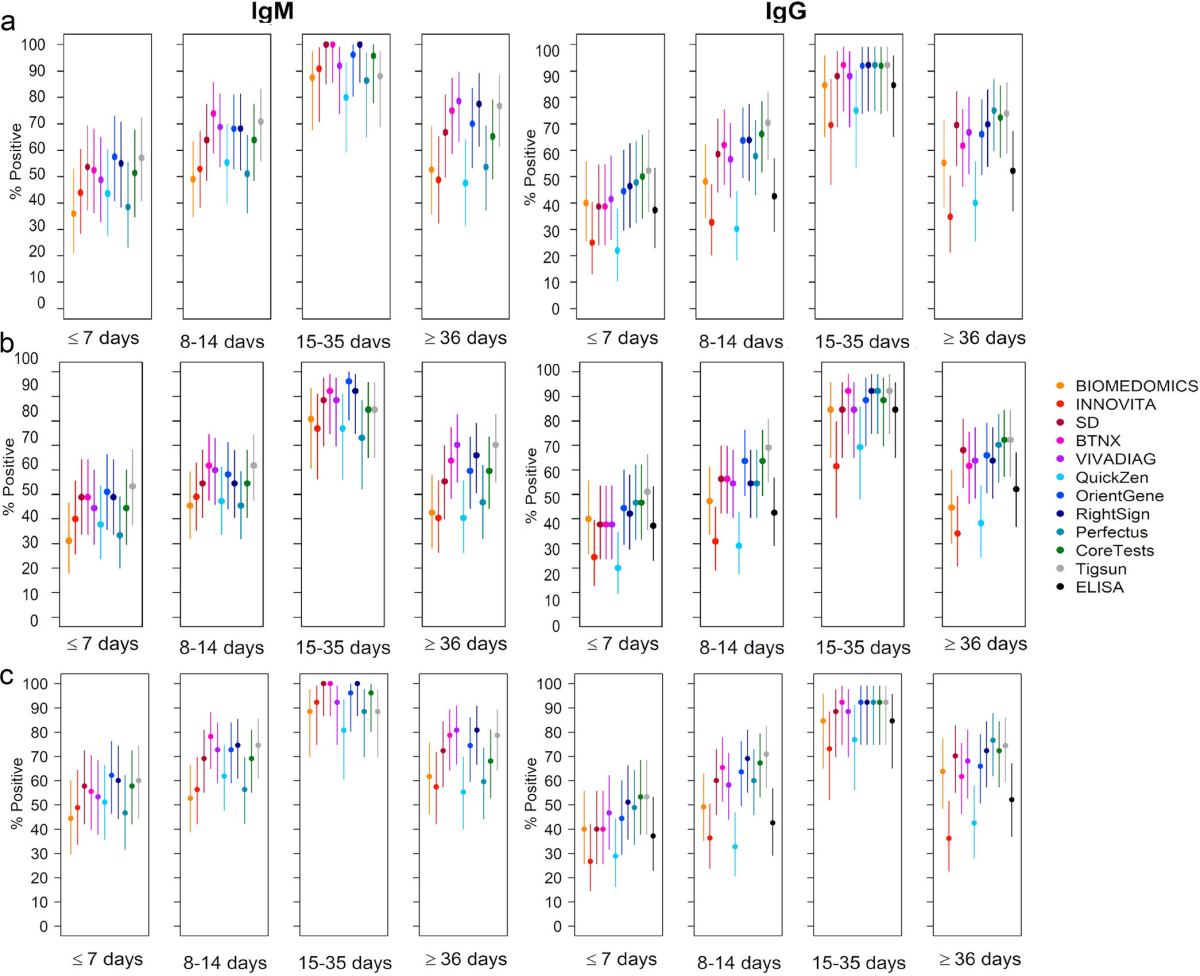
Sensitivity variations considering indeterminate results as positive (b) or negative (c). (a) Test sensitivity excluding indeterminate results plotted for IgM and IgG. For IgM, a significant difference emerged only between samples collected at ≤7 days after symptom onset and samples collected at 15 to 35 days (*P* = 0.0072). For IgG, a significant difference emerged between samples collected at ≤7 days after symptom onset and samples collected at 15 to 35 days (*P* = 0.0004) and samples collected at 8 to 14 days after symptom onset and samples collected at 15 to 35 days (*P* = 0.0188). (b) Sensitivity considering indeterminate results to be positive plotted for IgM and IgG. (c) Sensitivity considering indeterminate results to be negative plotted for IgM and IgG.

**FIG 4:**
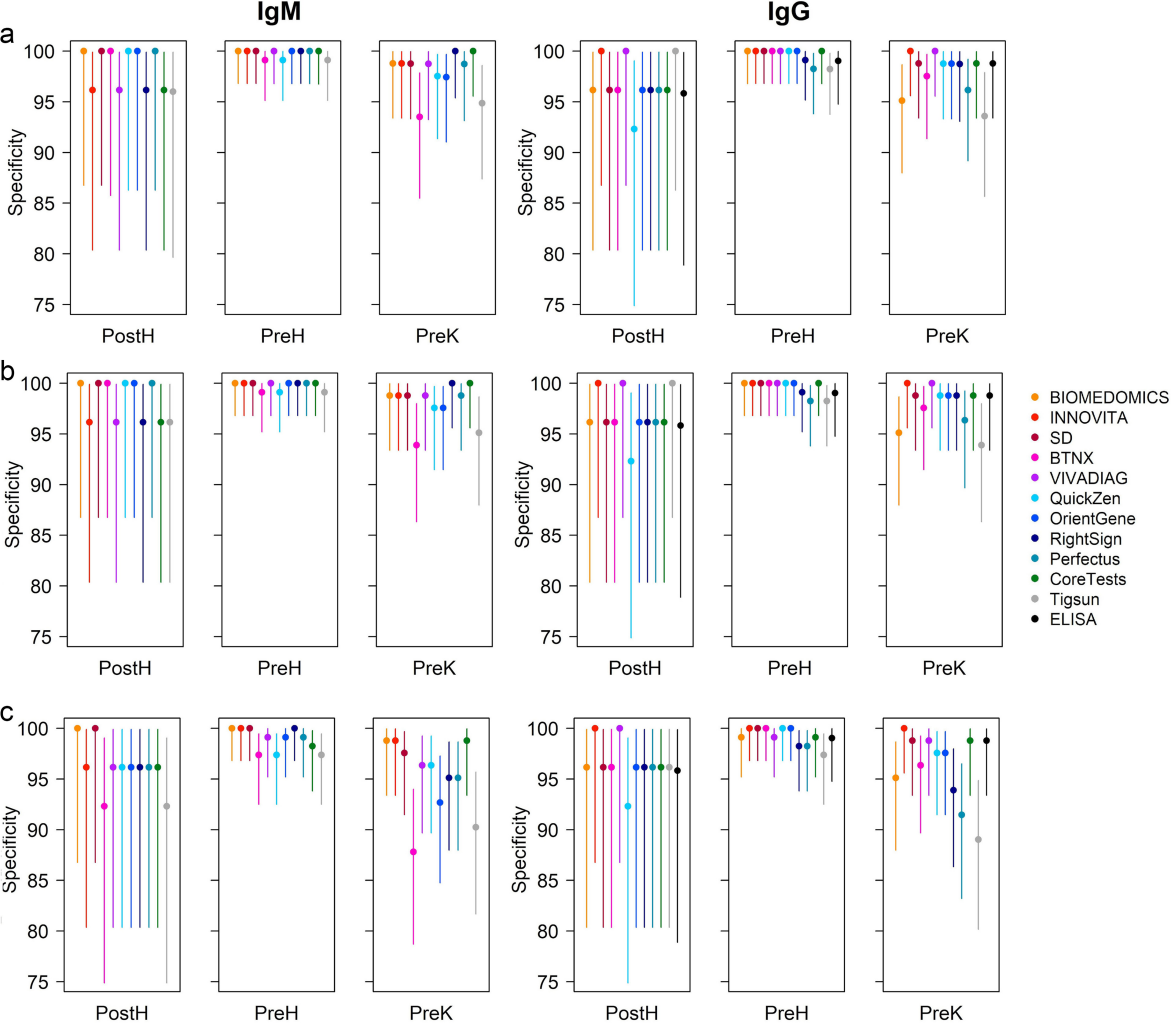
Specificity variations considering indeterminate results as positive (b) or negative (c). (a) Test specificity excluding indeterminate results plotted for IgM and IgG. For IgM, the results were significantly less likely to be negative in TB subjects (PreK) than in healthy subjects (PreH) (*P* value = 0.0115). For IgG, no statistically significant differences have been observed. (b) Specificity considering indeterminate results as positive plotted for IgM and IgG. (c) Specificity considering indeterminate results as negative plotted for IgM and IgG. PreH, *n* = 114; PreK, *n* = 82; PostH, *n* = 26. Error bars refer to 95% CIs.

Indeterminate results could be interpreted as positive or negative if an indeterminate cutoff is not clearly defined before proceeding with the exam. Therefore, it was evaluated if the capability of the tests for identifying true negative and true positive samples was affected if the indeterminate results were, respectively, considered all positive or negative. The indirect effects of these modifications on the sensitivity and specificity of each LFA were carefully examined (Fig. 3 and 4).

When comparing the obtained results to the sensitivity calculated excluding the indeterminate results, if considering the indeterminate results as positive, an increase in sensitivity from 2% to 8% was reported according to the test, and a loss in sensitivity of 2% to 7% was reported if considering them as negative (Fig. 3). Variations in specificity (Fig. 4) have also been reported, with a loss in specificity from 2% to 4% considering indeterminates as positive and an increase of 2% to 3% considering them as negative.

### Concordance between tests.

**FIG 5:**
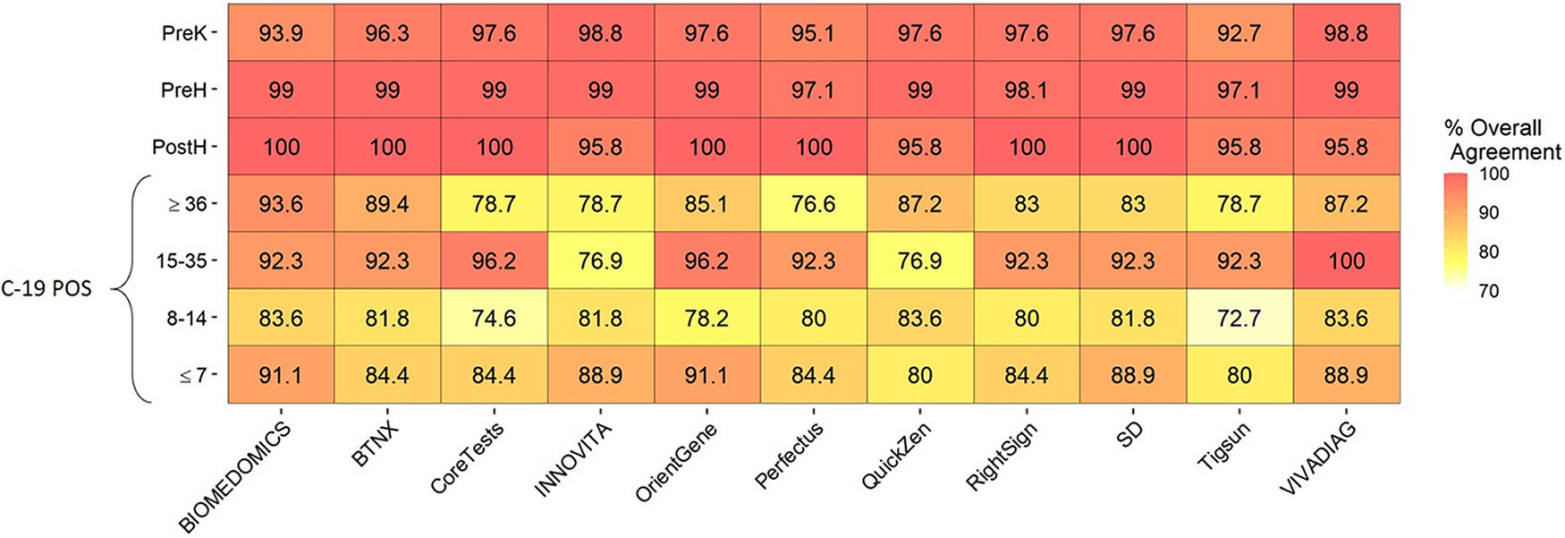
Concordance rate between ELISA and LFAs for IgG. The concordance rate between LFAs and ELISA in samples from COVID-19 patients remained at each time point higher than 70%. The highest agreement level with ELISA was observed between 15 and 28 days from symptom onset for all of the LFAs with the exclusion of BioMedomics, Innovita, and QuickZen. BioMedomics and QuickZen demonstrated 93.6% of agreement at more than 36 days. Innovita showed 88.9% of agreement at less than 7 days. The only test that reached 100% of concordance with ELISA was VivaDiag at 15 to 35 days from symptoms onset.

The agreement between LFAs and ELISA for IgG has been estimated (Fig. 5). The concordance level between LFAs and ELISA in the COVID-19-positive cohort remained at each time point higher than 70%, reaching 100% only for one test (VivaDiag) at one time point (15 to 35 days from symptom onset).

Overall, in the COVID-19-positive groups, the highest concordance rate was observed between 15 and 35 days from symptom onset (BTNX, Perfectus, RightSign, SD, and Tigsun, 92.3%; Orient Gene, 96.2%; VivaDiag, 100%). Only for two LFAs (BioMedomics and QuickZen) was the highest concordance rate with ELISA reached at ≥36 days from symptom onset (respectively, 93.6% and 87.2%).

For the PostH group, the results provided for IgG by BioMedomics, BTNX, Coretests, Orient Gene, Perfectus, RightSign, and SD correlated at 100% with ELISA results. In the PreH group, the maximum concordance rate between LFAs and ELISA was 99%. The agreement level was lower for the PreK cohort, reaching a peak of 98.8% for Innovita and VivaDiag.

The concordance rate between different LFAs plotted for IgM and IgG in PreH, PostH, and PreK groups is provided in Fig. S1A and S1B in the supplemental material. The overall level of agreement for IgM is higher than 92% in the PostH cohort and higher than 95% in the PreH one. In the PreK cohort, instead BTNX concordance rate with the other tests was low and reached its highest point at 87.8% with Orient Gene. Noticeably, BTNX was the only test that reported in its instruction for use that TB drugs (rifampicin, 78.1 μmol/liter; isoniazid, 292 μmol/liter; ethambutol, 58.7 μmol/liter) were tested as possible interfering substances without affecting the test’s performance. Nonetheless, for IgM in the PreK group, we observed a dip in BTNX specificity for IgM that drops under 95% (93.51%; 95% CI, 85.49 to 97.86).

### Seroconversion pattern.

An evaluation of the seroconversion pattern has been performed sampling 45 individuals at two different time points. Of 16 patients sampled at ≤7 days from symptom onset, 3 were reanalyzed between 15 and 35 days and 13 at ≥36 days; of the 23 patients firstly sampled between 8 and14 days, 3 were again collected between 15 and 35 days and 20 at ≥36 days, and finally 6 were sampled at 15 to 35 days and then at ≥36 days.

**FIG 6:**
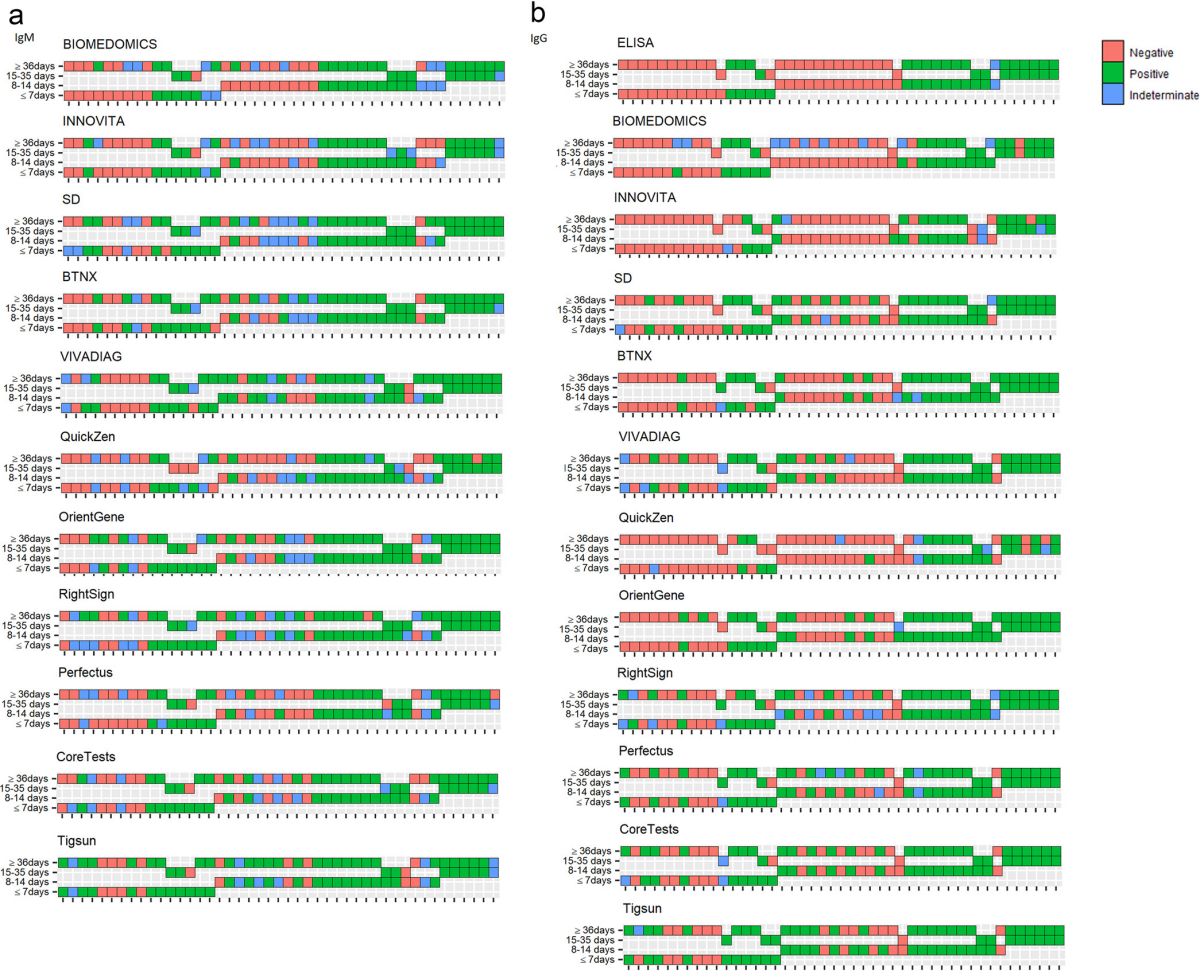
IgM and IgG evaluated at two different time points in 45 individuals. (a) IgM seroconversion pattern for each test. (b) IgG seroconversion pattern for each test. Forty-five individuals were sampled at two different time points. Each sample is represented by a square. Red identifies negative samples, blue identifies indeterminate ones, and green identifies positive ones. Samples from the same patients are represented on the same vertical axis. Sixteen patients were sampled at ≤7 days from symptom onset. Three of them were reanalyzed between 15 and 35 days from symptom onset, and 13 were reanalyzed at ≥36 days. Twenty-three patients were first sampled between 8 and 14 days. Three were examined a second time between 15 and 35 days and 20 at ≥36 days. Six patients were sampled between 15 and 35 days and then at ≥36 days.

Decay of the antibody responses have been reported for IgM starting from 23 days after symptom onset, while IgG titers appeared to be stable for up to 79 days (18). In our study, IgM seroreversion was observed within 30 days from symptom onset with BioMedomics, Innovita, QuickZen, Orient Gene, Perfectus, Coretests, and Tigsun (Fig. 6a). Moreover, IgG decay was observed for three samples with Innovita and two with QuickZen within 1 month of symptom onset. Interestingly, one patient that tested positive for IgG at ≤7 days from symptom onset resulted negative once retested 30 days after the first sampling with all of the LFAs in the study, apart from VivaDiag and Tigsun. For only this patient, the seroreversion observed with LFAs was confirmed with ELISA. The patient was a 61-year-old male with a history of hypertension and diabetes who developed acute respiratory distress syndrome (ARDS) during his hospitalization but was not admitted into the intensive care unit (ICU). The same patient tested positive for IgM with all LFAs in the study immediately after admission into hospital, but 30 days later, at the second sampling, an IgM decay was observed too with 7/11 LFAs. The other four tests provided an indeterminate result.

The occurrence of seroreversion for both IgG and IgM does not appear to be related to any specific preexisting condition or to the severity of the illness for any of the subject in the analysis.

### Health care workers.

Of the 57 health workers sampled at 21 to 24 days from the first dose of the Pfizer vaccine, all showed a positive titer of IgG against SARS-CoV-2 spike protein RBD (≥0.8 U/ml), detectable by ECLIA (Table 1). Of the 11 LFAs used to detect an IgM response, only two, Tigsun and BTNX, failed to show a positive response in any of the samples tested, while Orient Gene had an IgM positivity rate of 12.24% (6/49) and the highest IgG detection rate (85.71%; 48/56) (Table 2).

**TABLE 1:** Quantitative IgG ECLIA results compared with IgG positive or negative LFA identification in vaccinated health workers

Ab-COVID-Spike (ECLIA)	IgG negative to LFA				IgG positive to LFA			
	No.	Median (U/ml)	Minimum (U/ml)	Maximum (U/ml)	No.	Median (U/ml)	Minimum (U/ml)	Maximum (U/ml)
VivaDiag	35	54.1	1.57	>2,500				
Innovita	28	25.85	1.57	>2,500				
BTNX	6	5.49	1.57	11.5	8	66.95	15.4	229
BioMedomics	2	12.655	6.21	19.1	3	62	31.8	132
SD	13	16.5	1.57	107	35	122	5.27	>2,500
Coretests	34	20.45	1.57	213	17	197	5.27	>2,500
RightSign	21	17.2	1.57	107	22	181	8.15	>2,500
Perfectus	15	11.1	1.57	107	35	122	8.15	>2,500
QuickZen	26	19	1.57	222	17	193	11.5	>2,500
Orient Gene	7	9.74	1.83	107	47	54.1	4.04	>2,500
Tigsun	48	36.15	1.57	367	5	2,500	5.27	>2,500

**TABLE 2:** Rate of IgM and IgG that resulted positive, negative, or indeterminate at different LFAs in vaccinated health workers

Ig and test	Negative result		Positive result		Indeterminate	Invalid	Not assessed
	No.	%	No.	%			
IgM							
VivaDiag	36	97.3	1	2.7	0	1	20
Innovita	27	96.43	1	3.57	2	0	28
BTNX	18	100	0	0	2	0	38
BioMedomics	5	83.33	1	16.67	1	0	51
SD	54	96.43	2	3.57	2	0	0
Coretests	55	98.21	1	1.79	1	0	1
RightSign	52	92.86	4	7.14	1	0	1
Perfectus	52	94.55	3	5.45	2	0	1
QuickZen	44	91.67	4	8.33	8	0	2
Orient Gene	43	87.76	6	12.24	8	0	1
Tigsun	57	100	0	0	0	0	1
IgG							
VivaDiag	37	100	0	0	0	1	20
Innovita	30	100	0	0	0	0	28
BTNX	6	37.5	10	62.5	4	0	38
BioMedomics	2	33.33	4	66.67	1	0	51
SD	14	28	36	72	8	0	0
Coretests	36	67.92	17	32.08	4	0	1
RightSign	21	47.73	23	52.27	13	0	1
Perfectus	15	28.85	37	71.15	5	0	1
QuickZen	28	62.22	17	37.78	11	0	2
Orient Gene	8	14.29	48	85.71	1	0	1
Tigsun	50	90.91	5	9.09	2	0	1

The rate of positivity to IgG of the different LFAs was evaluated in comparison to the quantitative results obtained by ECLIA. As shown in Table 1, VivaDiag and Innovita did not detect positive IgG for ECLIA titers >2,500 U/ml. The lowest positive titer was correctly identified by Orient Gene (4.04 U/ml).

## DISCUSSION

In this study, we analyzed the performance of 11 different LFAs and one commercial ELISA in detecting SARS-CoV-2-specific IgG and IgM. Specificity was assessed in three cohorts as follows: historic samples collected before 2019 in healthy donors, in patients who were on TB treatment, and in individuals who tested RT-PCR negative for SARS-CoV-2.

The overall sensitivity of the tests (positive to IgM and/or IgG) was calculated by evaluating capability to correctly identify individuals confirmed to have been infected with SARS-CoV-2 by rRT-PCR. The obtained data allowed us to identify Coretests as the test with the highest specificity and sensitivity at ≥36 days from symptom onset. Hence, the latter appeared to be more appropriate for a serological mass screening due to the lower risk of identifying false positives because of the high specificity.

Furthermore, Orient Gene demonstrated the highest sensitivity in identifying a positive titer of IgG against protein S RBD, proving itself a possible test to evaluate an immunological response after the vaccine. Considering the limited number of cases analyzed, an in-depth evaluation on a wider cohort is needed to assess the effective reliability for this purpose of Orient Gene in comparison to other LFAs. Interestingly, VivaDiag and Innovita, even though they demonstrated good specificity (both 100%; 95% CI, respectively, 95.55 to 100 and 95.60 to 100) and sensitivity (VivaDiag, 94.12%; 95% CI, 71.31 to 99.85; Innovita, 86.67%, 95% CI, 59.54 to 98.34) in identifying positive subjects at 15 to 28 days from symptom occurrence, did not detect the IgG response at 21 days from the vaccination. Indeed, more information by LFA manufacturers on the antigenic targets of their tests would help to perform a more on-point evaluation of these tests. A further study, including more time points from symptom onset, could be useful to evaluate the reliability of LFAs to identify a previous infection of SARS-CoV-2 at 60 and 90 days from the infection. The main limitation in the sensitivity assessment is due to the lack of asymptomatic and paucisymptomatic patients in our cohort to perform an evaluation of the rate of positivity in association with the severity of the developed disease.

The cohort of patients in therapy for TB was included to evaluate the possible effects of TB drugs on LFA performance, since one test (BTNX) recognized in its product insert rifampicin, ethambutol, and isoniazid as possible interfering substances. The manufacturers declared that sensitivity and specificity of the BTNX test were not affected by the presence of these drugs at therapeutic concentrations in blood. Nonetheless, the level of agreement of BTNX with other LFAs for IgM is the lowest in the PreK group; therefore, TB therapy could have had an effect on the test despite what is declared by the manufacturer. Even if it is well known that rifampicin can cause false-positive immunoassay results for urine opiates (19), to our knowledge, this is the first report that provides proof that TB medicines can affect SARS-CoV-2 LFAs for antibody detection. This occurrence probably deserves an in-depth analysis to identify the possible mechanisms for cross-reactivity, but it is to be kept in account if LFAs will be used in countries with a high TB prevalence.

A higher number of indeterminate results was overall observed for IgM than for IgG. The identification of these faint bands affected the general efficiency and reliability mainly of two of the LFAs in analysis, BTNX and VivaDiag. The repetition of the indeterminate exams did not provide a clear positive or negative result in the majority of cases for BTNX IgM, as 30/395 still remained not interpretable. Previous studies have suggested that all faint identified bands should be considered negative to improve the specificity of the test (10). However, our analysis demonstrated that considering negative all of the samples identified as indeterminate would result in a major decrease in the sensitivity of the tests (up to 7%) compared with a minimal gain in specificity (2% to 3%). Moreover, the definition of “faint” is based on a subjective evaluation of the band intensity that in future may be objectified through the use of automatic readers or, at least, through an attentive training of the readers (20).

In conclusion, the tests analyzed demonstrated different performances and different levels of reliability in identifying IgM and IgG against SARS-CoV-2. Therefore, great prudence should be used to employ the most accurate point-of-care (POC) serological tests to evaluate local epidemiology as well as to verify the development of an immunological response after the vaccine, especially in diagnostically challenging settings. The need for reader training as well as the possible interference of TB therapy on the tests results have been identified by our study as two of the main limiting factors for the use of these tests in low-middle-income countries. Finally, in a period of scarcity of vaccine doses, when several European countries, including Italy and France, are recommending a single dose of vaccine for individuals who were positive for SARS-CoV-2 in the previous 6 months, the tests with the highest specificity may be used to determine a prior infection and therefore deeply influence the vaccination campaign (3, 4).

## MATERIALS AND METHODS

### Study design: setting and population.

**FIG 7:**
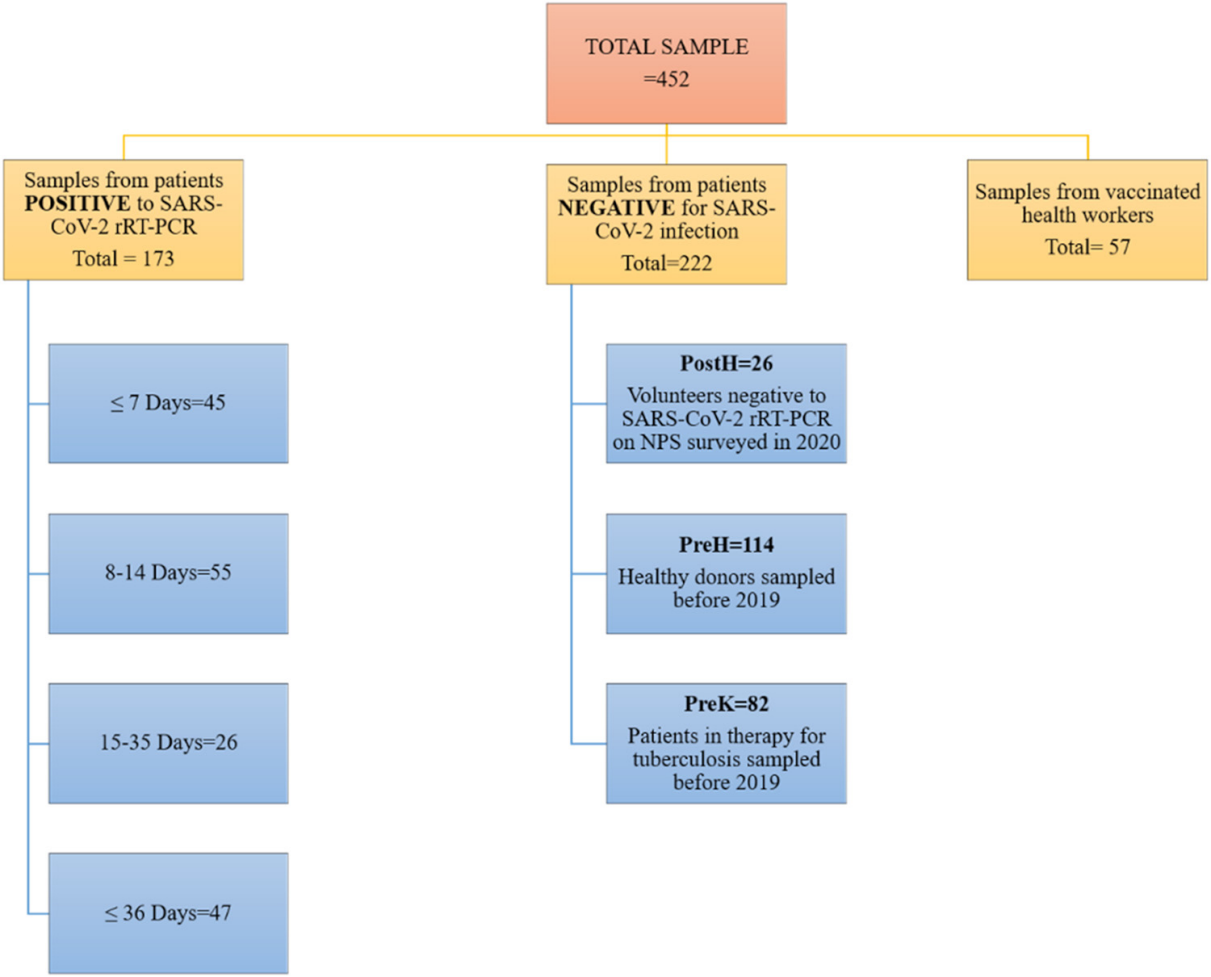
Flow chart of the study sample and population definition.

This study included two different sampling phases that took place, respectively, between April and June 2020 and January and February 2021 at San Raffaele Research Hospital in Milan, Italy. The different patient cohorts analyzed are summarized in Fig. 7.

During the first phase, in the spring of 2020, 128 symptomatic COVID-19 patients who resulted positive with rRT-PCR performed on nasopharyngeal swab (NPS) participated in the study, and from 45 of them, two samples were collected at different time points. All patients were hospitalized. Their symptoms included cough (58.13%), dyspnea (55.40%), and fever (89.01%). Moreover, 49.71% of the patients developed acute respiratory distress syndrome (ARDS), and 16.18% died. None of the COVID-19 patients had a history of chronic obstructive pulmonary disease (COPD), but other chronic diseases such as diabetes and hypertension were reported (respectively, 9.82% and 32.94%). All clinical data were extracted from the San Raffaele Research Hospital internal database. At the same time point, 26 plasma samples were collected from volunteers who tested negative for SARS-CoV-2 infection by rRT-PCR performed on NPS (designated in tables and figures as the PostH group).

To evaluate the specificity of the tests, 196 samples collected and stored before 2019 were included in the analysis; 82 were from patients in therapy for tuberculosis (TB), and 114 were from healthy donors (in tables and figures, they are referred to as the PreK group and PreH group, respectively). The patients in therapy for TB have been included to evaluate any possible interfering effect of TB drugs on test specificity.

All samples were collected by venipuncture in serum tubes with spray-coated silica or in K2EDTA tubes, stored at +4°C and aliquoted for freezing at −80°C within 1 week of the blood draw. Serum and plasma were used interchangeably for all tests, except for Euroimmun ELISA applicable only with serum samples.

The clinical and demographic characteristics of this population are summarized in Tables 3 and 4.

**TABLE 3:** Demographics and clinical characteristics of COVID-19-positive population

Variable	Category	COVID-19 POS at ≤7 days (*n* = 45)		COVID-19 POS at 8 to 14 days (*n* = 55)		COVID-19 POS at 15 to 35 days (*n* = 26)		COVID-19 POS at day 36+ (*n* = 47)	
		No.	%	No.	%	No.	%	No.	%
Sex	Male	28	62.22	38	69.09	19	73.08	33	70.21
	Female	17	37.78	17	30.91	7	26.92	14	29.79
	NA^*a*^	0	NA	0	NA	0	NA	0	NA
Ethnic group	Caucasian	36	80	45	81.82	22	84.62	38	80.85
	Hispanic	5	11.11	8	14.55	4	15.38	6	12.77
	Asian	2	4.44	1	1.82	0	0	1	2.13
	Black	2	4.44	1	1.82	0	0	2	4.26
	NA	0	NA	0	NA	0	NA	0	NA
Hypertension	no	29	64.44	35	63.64	17	65.38	35	74.47
	yes	16	35.56	20	36.36	9	34.62	12	25.53
	NA	0	NA	0	NA	0	NA	0	NA
Coronary artery disease	no	42	93.33	48	87.27	25	96.15	46	97.87
	yes	3	6.67	7	12.73	1	3.85	1	2.13
	NA	0	NA	0	NA	0	NA	0	NA
Diabetes	no	35	77.78	44	80	20	76.92	43	91.49
	yes	4	8.89	5	9.09	5	19.23	3	6.38
	NA	6	13.33	6	10.91	1	3.85	1	2.13
COPD	no	45	100	55	100	26	100	47	100
	yes	0	0	0	0	0	0	0	0
	NA	0	NA	0	NA	0	NA	0	NA
Neoplasia	no	44	97.78	53	96.36	26	100	47	100
	yes	1	2.22	2	3.64	0	0	0	0
	NA	0	NA	0	NA	0	NA	0	NA
Cough	no	22	48.89	22	40.00	10	38.46	18	38.3
	yes	23	51.11	32	58.18	16	61.54	29	61.7
	NA	0	0	1	1.82	0	0	0	0
Dyspnea	no	19	42.22	21	38.18	13	50	24	51.06
	yes	26	57.78	34	61.82	13	50	23	48.94
	NA	0	NA	0	NA	0	NA	0	NA
Fever	no	7	15.56	5	9.09	3	11.54	4	8.51
	yes	38	84.44	50	90.91	23	88.46	43	91.49
	NA	0	NA	0	NA	0	NA	0	NA
Nausea, vomiting, and/or diarrhea	no	41	91.11	47	85.45	24	92.31	44	93.62
	yes	4	8.89	7	12.73	2	7.69	3	6.38
	NA	0	0	1	1.82	0	0	0	0
Headache	no	44	97.78	51	92.73	25	96.15	44	93.62
	yes	1	2.22	3	5.45	1	3.85	3	6.38
	NA	0	0	1	1.82	0	0	0	0
Syncope	no	42	93.33	52	94.55	26	100	46	97.87
	yes	3	6.67	2	3.64	0	0	1	2.13
	NA	0	0	1	1.82	0	0	0	0
Ageusia-anosmia	no	43	95.56	53	96.36	26	100	46	97.87
	yes	2	4.44	1	1.82	0	0	1	2.13
	NA	0	0	1	1.82	0	0	0	0
Myalgia-arthralgia	no	44	97.78	50	90.91	26	100	45	95.74
	yes	1	2.22	4	7.27	0	0	2	4.26
	NA	0	0	1	1.82	0	0	0	0
Chest pain	no	38	84.44	53	96.36	26	100	45	95.74
	yes	7	15.56	1	1.82	0	0	2	4.26
	NA	0	0	1	1.82	0	0	0	0
ARDS	no	19	42.22	21	38.18	10	38.46	21	44.68
	yes	23	51.11	30	54.55	10	38.46	23	48.94
	NA	3	6.67	4	7.27	6	23.08	3	6.38
Death	no	34	75.56	42	76.36	25	96.15	44	93.62
	yes	11	24.44	13	23.64	1	3.85	3	6.38
	NA	0	NA	0	NA	0	NA	0	NA
ICU	no	31	68.89	34	61.82	15	57.69	37	78.72
	yes	14	31.11	21	38.18	11	42.31	10	21.28
	NA	0	NA	0	NA	0	NA	0	NA

a NA, not available.

**TABLE 4:** Demographics of the COVID-19-negative population

Variable	PostH (*n* = 26)	PreH (*n* = 114)	PreK (*n* = 82)
Male sex (no. [%])	11/26 (42.31)	49/112 (43.75)	57/80 (71.25)
Ethnic group (no. [%])			
Caucasian	26/26 (100)	114/114 (100)	32/78 (41.03)
Hispanic	0	0	3/78 (3.85)
Asian	0	0	12/78 (15.38)
Black	0	0	31/78 (39.74)
Age (median [IQR^*a*^])	34 (32, 41)	22 (21, 23)	30 (23, 48)

a IQR, interquartile range.

To evaluate the capability of the tests for identifying an immune response elicited by the COVID-19 vaccine, a cohort of 58 health workers who had received the first dose of BNT162b2 Pfizer-BioNTech vaccine 21 to 24 days before was surveyed between January and February 2021. One clotted sample was finally excluded from the analysis. They were all adults, and females and males were equally represented. None of them reported any relevant comorbidity or previous immunological disease or allergic reaction to drugs and/or vaccines. From this cohort, all samples were collected by venipuncture, stored at +4°C and analyzed in the 24 h following the collection using 11 different LFAs and an ECLIA, dosing IgG against Spike protein RBD.

### Immunochromatographic LFAs.

Eleven LFAs were utilized according to the manufacturer’s instructions (see Table S1 in the supplemental material).

In brief, the appropriate sample volume was added on the indicated sample port, followed by a defined amount of the provided diluent. The cartridges were then incubated at room temperature for the recommended time. Result reading was performed by two independent observers. In case of disagreement, a third reader was consulted, and the final result was given by two concordant readings.

Samples were considered negative if the control band was present and the test band absent and positive if both the bands were clearly observed. An indeterminate result was given if the control band has been identified jointly to a faint test band, with an intensity definitely lower than the control one and that could not be clearly associated to a positive reaction. The test was considered invalid if the control band was not identified.

All indeterminate and invalid results were repeated once. If a clear interpretation of the test was still not possible because of the presence of a low-intensity band identified by both readers, the test result was confirmed as indeterminate. None of the samples tested invalid a second time for any of the tests in analysis.

### ELISA.

Euroimmun anti-SARS-CoV-2 ELISA for the detection of IgG against the SARS-CoV-2 S1 domain was carried out according to the manufacturer’s instructions. In brief, 10 μl of serum was diluted 1:101 in the provided sample buffer. Then 100 μl of the diluted samples, calibrator, and positive and negative controls were transferred into the precoated microplate wells according to the provided pipetting protocol and incubated at 37°C for 60 min. Following the washing step, conjugate and then substrate incubations were performed before the addition of the stopping solution and the consequent photometric measurement.

### ECLIA.

Elecsys anti-SARS-CoV-2 S (Roche) is an ECLIA for the determination of IgG against the SARS-CoV-2 spike (S) protein receptor binding domain (RBD). The assay, based on a double-antigen sandwich assay format, has been performed according to the manufacturer’s instructions on a cobas e 411 analyzer on the 57 samples collected from health workers after the first vaccination dose.

### Statistical analysis.

Descriptive statistics of continuous variables were presented as median and interquartile range, while for categorical variables, frequencies were reported. In the absence of a gold-standard test for serology detection, sensitivity and specificity were estimated using surrogate reference standards. Sensitivity was estimated using samples collected from patients confirmed by rRT-PCR to have been infected with SARS-CoV-2, while specificity was estimated using samples from healthy negative controls and patients receiving therapy for tuberculosis collected prior to the circulation of SARS-CoV-2. Binomial exact 95% confidence intervals were calculated for all estimates. Logistic mixed-effects models were used to evaluate differences among groups, since the data consist of repeated measurements of the same subjects. The agreement between assays was evaluated by computing the percentage of concordant results. All statistical analyses were performed using R statistical software version 4.0.4 (www.r-project.org).

### Ethical approval.

This study was approved by the ethical committee and institutional review board of San Raffaele Research Hospital in Milan, Italy (protocol number COVID-19 IA evaluation). All patients and healthy controls agreed to the study by signing the informed consent.
