# Supplementary material for: SARS-CoV-2 Antibody Rapid Tests: Valuable Epidemiological Tools in Challenging Settings
Source: Microbiol Spectr. 2021 Sep 22;9(2):e00250-21. doi: 10.1128/Spectrum.00250-21 (PMC8557824; doi:10.1128/Spectrum.00250-21)
Supplement: SUPPLEMENTAL FILE 1 — Supplemental material. Download SPECTRUM00250-21_Supp_1_seq3.pdf, PDF file, 0.4 MB [file spectrum00250-21_supp_1_seq3.pdf]

SUPPLEMENTARY MATERIAL:

Table S1. LFAs manufacturers and Antigenic target.

Name	Manufacturer	Ag Target
COVID-19 IgM/IgG Rapid Test	Biomedomics	Undisclosed
2019-nCoV Ab Test	Innovita	S1+N
STANDARD Q COVID-19 IgM/IgG Duo	SD Biosensor	N
COVID-19 IgG/IgM Test	BTNX	Undisclosed
COVID19 Single Rapid Test IgM/IgG	VivaDiag	Undisclosed
COVID-19 IgM/IgG	QuickZen	Undisclosed
COVID-19 IgG/IgM Rapid Test	OrientGene	Undisclosed
COVID-19 IgG/IgM Rapid Test Cassette	RightSign	Undisclosed
Novel Corona Virus (SARS-CoV-2) IgM/IgG Rapid Test Kit	Bio Perfectus	Undisclosed
COVID-19 IgM/IgG Ab Test	CoreTests, Core Technology	Undisclosed
COVID-19 Combo IgM/IgG Rapid Test (Lateral Flow Method)	Tigsun	Undisclosed
Anti-SARS-CoV-2 ELISA IgG	Euroimmun	S1

Figure S1. Agreement of LFAs for SARS-CoV-2

Percent agreement plotted per IgM (a) and IgG (b) across all LFAs combinations in PostH, PreH and PreK groups.

a)

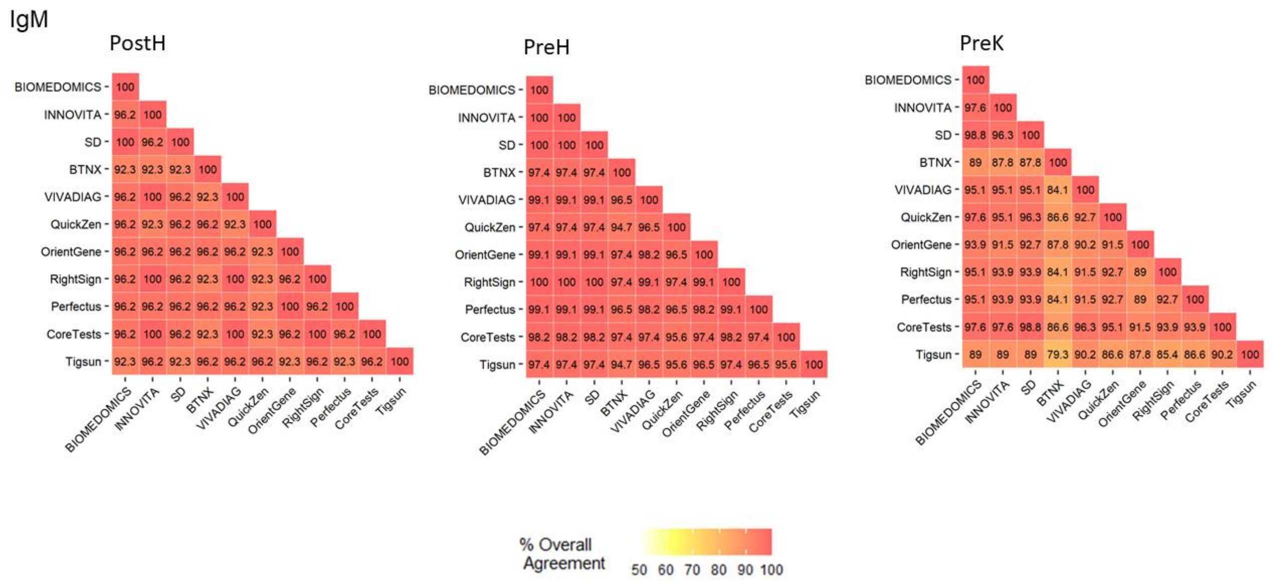

b)

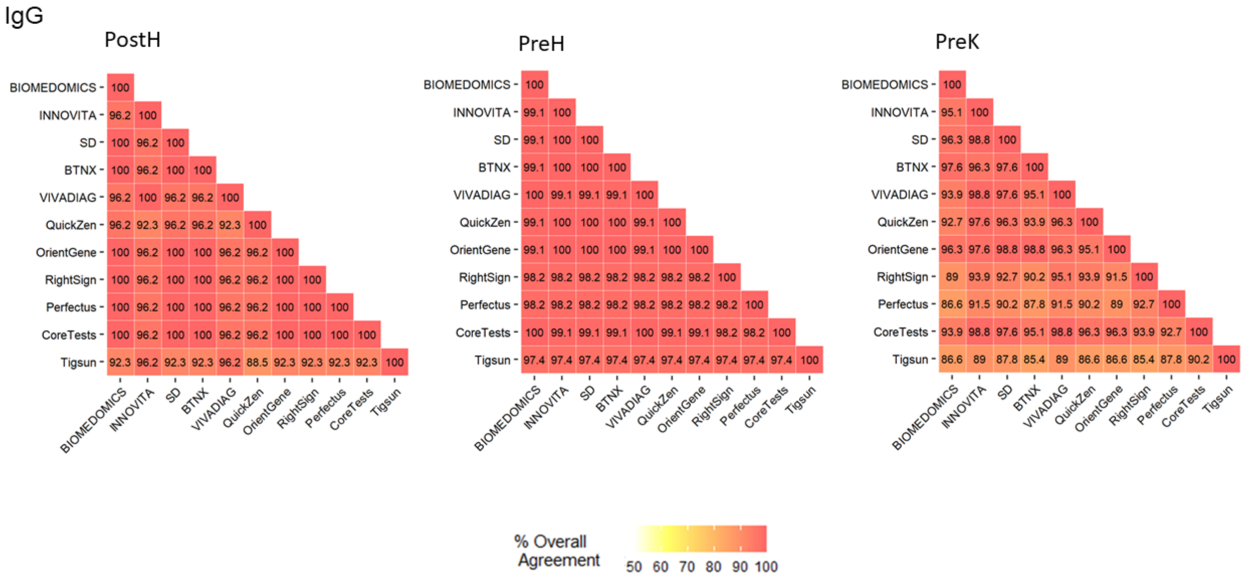

Table S2. Logistic mixed-effects model for IgM negative results

Parameter	Estimate	Std.Error	p-value
Intercept	7.0851	0.8910	<0.0001
Group (ref=PreH)			
PostH	-1.4251	1.1677	0.2223
PreK	-2.1369	0.8455	0.0115
Age	-0.0106	0.0199	0.5950
Female (ref=Male)	-0.9318	0.6395	0.1451

Table S3. Logistic mixed-effects model for IgG negative results

Parameter	Estimate	Std.Error	p-value
Intercept	5.2364	0.9569	<0.0001
Group (ref=PreH)			
PostH	-1.5841	1.3705	0.2477
PreK	-1.3598	0.8801	0.1223
Age	0.0161	0.0294	0.5854
Female (ref=Male)	2.4661	1.2573	0.0498

Table S4. Logistic mixed-effect models for IgM positive results and post-hoc pairwise comparisons

Parameter	Estimate	Std.Error	p-value
Intercept	-3.6571	1.8430	0.0472
Group (ref= C-19 POS ≤7 days)			
C-19 POS 8-14 days	0.8031	0.8131	0.3233
C-19 POS 15-35 days	3.3475	1.0330	0.0012
C-19 POS ≥36 days	1.1927	0.8552	0.1631
Age	0.0488	0.0290	0.0929
Female (ref=Male)	-0.3845	0.7311	0.5989

Ethnic group (ref=Caucasian)			
Hispanic	1.1459	0.9505	0.2280
Asian	0.6764	2.0802	0.7451
Afro-american	1.0614	1.8280	0.5615
ICU (ref=no ICU)	0.4117	0.7098	0.5619
**Post-hoc pairwise comparisons**			
**Contrast**			**p-value**
(C-19 POS - ≤7 days) - (C-19 POS - 8-14 days)			1
**(C-19 POS - ≤7 days) - (C-19 POS - 15-35 days)**			**0.0072**
(C-19 POS - ≤7 days) - (C-19 POS - ≥36 days)			0.9788
(C-19 POS - 8-14 days) - (C-19 POS - 15-35 days)			0.0613
(C-19 POS - 8-14 days) - (C-19 POS - ≥36 days)			1
(C-19 POS - 15-35 days) - (C-19 POS - ≥36 days)			0.2193

Table S5. Logistic mixed-effects model for IgG positive results and post-hoc pairwise comparisons

Parameter	Estimate	Std.Error	p-value
Intercept	-4.4812	1.5374	0.0036
Group (ref= C-19 POS ≤7 days)			
C-19 POS 8-14 days	1.0193	0.6826	0.1354
C-19 POS 15-35 days	3.4660	0.8695	0.0001
C-19 POS ≥36 days	1.5237	0.7222	0.0349
Age	0.0519	0.0242	0.0321
Female (ref=Male)	-0.0838	0.6057	0.8900
Ethnic group (ref=Caucasian)			
Hispanic	1.5409	0.7927	0.0519
Asian	1.5584	1.7520	0.3737
Afro-american	0.3960	1.5675	0.8006
ICU (ref=no ICU)	0.7769	0.5965	0.1928
**Post-hoc pairwise comparisons**			
**Contrast**			**p-value**
(C-19 POS - ≤7 days) - (C-19 POS - 8-14 days)			0.8122
**(C-19 POS - ≤7 days) - (C-19 POS - 15-35 days)**			**0.0004**
(C-19 POS - ≤7 days) - (C-19 POS - ≥36 days)			0.2092
**(C-19 POS - 8-14 days) - (C-19 POS - 15-35 days)**			**0.0188**
(C-19 POS - 8-14 days) - (C-19 POS - ≥36 days)			1
(C-19 POS - 15-35 days) - (C-19 POS - ≥36 days)			0.1484
